# AP-SKAT: highly-efficient genome-wide rare variant association test

**DOI:** 10.1186/s12864-016-3094-3

**Published:** 2016-09-21

**Authors:** Takanori Hasegawa, Kaname Kojima, Yosuke Kawai, Kazuharu Misawa, Takahiro Mimori, Masao Nagasaki

**Affiliations:** Department of Integrative Genomics, Tohoku Medical Megabank Organization, Tohoku University, 2-1 Seiryo-machi, Aoba-ku, Sendai, Miyagi, Japan

**Keywords:** Genome wide association study, Multiple test, Rare variants

## Abstract

**Background:**

Genome-wide association studies have revealed associations between single-nucleotide polymorphisms (SNPs) and phenotypes such as disease symptoms and drug tolerance. To address the small sample size for rare variants, association studies tend to group gene or pathway level variants and evaluate the effect on the set of variants. One of such strategies, known as the sequential kernel association test (SKAT), is a widely used collapsing method. However, the reported *p*-values from SKAT tend to be biased because the asymptotic property of the statistic is used to calculate the *p*-value. Although this bias can be corrected by applying permutation procedures for the test statistics, the computational cost of obtaining *p*-values with high resolution is prohibitive.

**Results:**

To address this problem, we devise an adaptive SKAT procedure termed AP-SKAT that efficiently classifies significant SNP sets and ranks them according to the permuted *p*-values. Our procedure adaptively stops the permutation test when the significance level is outside some confidence interval of the estimated *p*-value for a binomial distribution. To evaluate the performance, we first compare the power and sample size calculation and the type I error rates estimate of SKAT, SKAT-O, and the proposed procedure using genotype data in the SKAT R package and from 1000 Genome Project. Through computational experiments using whole genome sequencing and SNP array data, we show that our proposed procedure is highly efficient and has comparable accuracy to the standard procedure.

**Conclusions:**

For several types of genetic data, the developed procedure could achieve competitive power and sample size under small and large sample size conditions with controlling considerable type I error rates, and estimate *p*-values of significant SNP sets that are consistent with those estimated by the standard permutation test within a realistic time. This demonstrates that the procedure is sufficiently powerful for recent whole genome sequencing and SNP array data with increasing numbers of phenotypes. Additionally, this procedure can be used in other association tests by employing alternative methods to calculate the statistics.

## Background

High-throughput sequencing (HTS) technologies enable the detection of rare and common variants at the genome-wide scale for thousands of individuals [1, 2]. In addition, with population-specific reference panels comprised of detected variants from HTS, low-frequency variants can be imputed accurately from single-nucleotide polymorphism (SNP) array genotype data [3]. Thus far, associations between SNPs and disease phenotypes have been studied for genotype data from HTS and SNP arrays, and the recent focus has moved to rare and low-frequency variants. Unlike common variants, the power of rare and low-frequency variants on single-variant association tests is low because of the lack of allele counts, even with thousands of individuals.

To address this issue, rare and low-frequency variants are often grouped at the gene or pathway level, and the effects of multiple variants are evaluated. This type of strategy is called collapsing, and the sequential kernel association test (SKAT) [4, 5] is one of the most effective collapsing methods [6, 7]. Because the *p*-values based on SKAT are derived from an asymptotic distribution of its statistics, the *p*-values for datasets with an insufficient number of samples may be inaccurate, which causes inflation or power loss. To obtain accurate *p*-values, resampling methods such as the permutation test can be implemented in SKAT. However, resampling requires a huge amount of computation time to obtain high-resolution *p*-values for the correction of multiple comparisons, and hence a more efficient resampling method is necessary.

Therefore, we propose an adaptive procedure, termed AP-SKAT, for the highly efficient calculation of SKAT statistics. This procedure adaptively stops the permutation test when the significance level is outside some predetermined confidence interval for the estimated *p*-value. In this evaluation, we propose the following criteria to stop the permutation test and obtain a *p*-value: (i) when all permutation statistics are greater or less than the original statistic, the calculation is terminated when the probability of the event is less than the significance level, and (ii) the calculation is terminated when the confidence interval of the estimated *p*-value does not include a significance level. To show the effectiveness of the proposed procedure, we first evaluate the power and sample size calculations of SKAT [4], SKAT-O [5], and the proposed procedure using a genotype dataset in the SKAT R package [8]. Second, we also evaluate the type I error rate of SKAT-O and the proposed procedure using real whole genome sequencing (WGS) data from the 1000 Genomes Project (1000GP) [9]. Finally, computational experiments additionally using SNP array data downloaded from the Wellcome Trust Case Control Consortium (WTCCC) [10] and the International HapMap Project [11] show that the proposed procedure can calculate highly accurate *p*-values within a reasonable time. We conclude that the proposed procedure is applicable to recent sequencing and genotype imputed data with large amounts of phenotype data.

## Implementation

### Sequential kernel association test

Let *n* and *m* be the number of individuals and grouped SNPs, respectively. A SKAT test statistic *s* is calculated as

1 $$\begin{array}{*{20}l} s=(\boldsymbol{y} - \boldsymbol{\mu})^{\prime} GWG^{\prime} (\boldsymbol{y} - \boldsymbol{\mu}), \end{array} $$

where ***y*** is an *n*-dimensional vector of observed phenotypes, ***μ*** is an *n*-dimensional vector of predicted means under the null hypothesis, *i.e.*, the target phenotype has no association with the genotypes, using the logistic and the linear models for case/control studies and quantitative trait analysis, respectively. *G* is given by (***g***_1_,…,***g***_*i*_,…,***g***_*m*_)^′^, where ***g***_*i*_ is an *n*-dimensional vector including the genotypes of *n* individuals for the *i*th SNP and *W*=diag(*w*_1_,…,*w*_*j*_,…,*w*_*m*_) is an *m*×*m* diagonal matrix consisting of weights *w*_*j*_ for the *j*th variant.

In calculating SKAT statistics, we assume

2 $$\begin{array}{*{20}l} y_{i} = \alpha_{i} + \beta_{1}G_{1,i} + \ldots+ \beta_{m} G_{m,i} + \epsilon_{i},  \end{array} $$

where *y*_*i*_ is the *i*th element of ***y***,*α* is a constant that is unrelated to genotypes, *β*_*j*_ is the effect size of the *j*th SNP, *G*_*i*,*j*_ is the *i*th row and *j*th column of *G*, and *ε*_*i*_ is a noise term that obeys a Gaussian distribution. A good property of *s* is that it corresponds to a mixture of chi-squared distributions, and we can calculate the *p*-values for the obtained statistics when the optimal conditions are satisfied [4]. However, it has been suggested that the distribution of *s* differs from the ideal one when the sample size *n* is insufficient and the phenotype data do not follow a Gaussian distribution. Thus, in case/control or cohort genome studies with limited samples, it is not valid to evaluate the test statistics based on a mixture of chi-squared distributions. In this case, Lee et al. [5] suggested to use the optimal adjustment technique termed SKAT-O to combine burden test and the moment adjustment technique to modify the distribution instead of using the permutation test, and Wu et al. [12] also proposed an alternative calculation procedure to efficiently and analytically calculate the adaptive sum of SKAT-O statistics. However, even when applying these techniques, the modified distribution includes residual biases. Additionally, for the permutation test with more than 20,000 SNP sets, grouping SNPs into gene level and considering multiple test is not practical because it requires at least 4.0×10^5^ (*α*_*p*_=5.0×10^−2^) or 2.0×10^6^ (*α*_*p*_=1.0×10^−2^) tests for each SNP set, where *α*_*p*_ is the significance level. Thus, we focus on obtaining detailed *p*-values for sets of rare SNPs associated with phenotypes around the predefined significance level *α*_*p*_ through the permutation test, and efficiently calculate *p*-values by adaptively stopping the test for plausible/improbable sets.

### Distribution of estimated *p*-values in permutation test

In the process of a permutation test, let *B* and *r* be the number of permutations completed and the number of permutation statistics that are greater than the original statistic *s* using the observed data, respectively. In this case, we consider a binary random variable *X*, which takes a value of 1 when a permutation statistic is greater than *s* and 0 otherwise, according to a previous SNP analysis [13]. We take the expectation and the variance of *X* corresponding to each of the permutations considered so far to be

3 $$\begin{array}{*{20}l} \text{Exp}[X] &= \hat{p}=r/B, \end{array} $$

4 $$\begin{array}{*{20}l} \text{Var}[X] &= \text{Exp}[X^{2}] - \text{Exp}[X]^{2}=\hat{p}(1-\hat{p}), \end{array} $$

where $\hat {p}$ is the estimated *p*-value of an SNP set on the *B*th permutation. Thus, the Bienaymé formula for the sum of variances gives the variance of the mean as

5 $$\begin{array}{*{20}l} \text{Var}[\hat{p}] = \frac{\hat{p}(1-\hat{p})}{B}. \end{array} $$

**Fig. 1:**
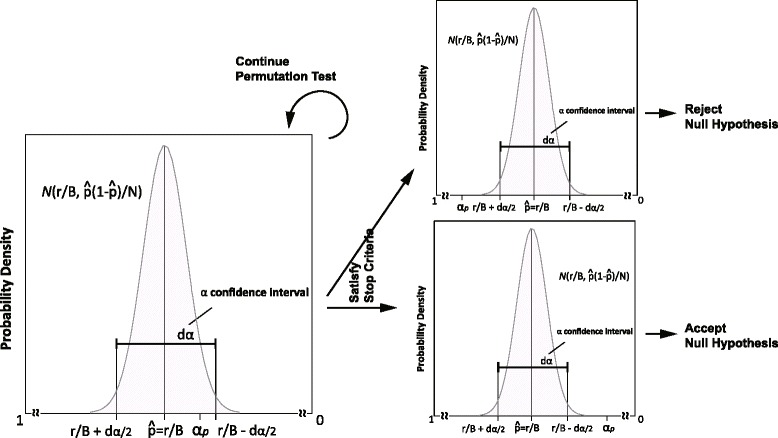
A sample figure exemplifying the distribution of the expectation of the estimated *p*-value and the stop criteria in the proposed procedure. The *α* confidence interval *d*_*α*_ of the distribution of the estimated *p*-value $\hat {p}$ is colored *gray*. *B* and *r* are the number of permutations completed and the number of permutation statistics that are greater than the original statistic *s* using the observed data, respectively. The stop criterion is evaluated using *p*±*d*_*α*_/2 and *α*_*p*_, which is the predefined significance level

According to the central limit theorem, we consider $\hat {p}$ to correspond to a Gaussian distribution $N(0, \hat {p}(1-\hat {p})/B)$ and obtain *d*_*α*_ as the distance between the *α* confidence interval of the distribution. In this binomial setting, we fix the number of permutations *B* and consider the numerator *r* as a random variable to estimate $\hat {p}$. Then, we compare the *α* confidence interval of $\hat {p}$ with *α*_*p*_, where *α*_*p*_ is a predetermined significance level considering multiple comparisons, and continue the permutation until either the *α* confidence interval does not include *α*_*p*_ or *B* becomes *b*. Figure 1 exemplifies this situation. In contrast, Chen et al. [13] used a negative binomial setting by fixing the total number of successes *r* and considering the denominator *B* as a random variable to estimate $\hat {p}$. They chose *b* and *R* to control the standard error of $\hat {p}$ at some determined values with *α*_*p*_, and continued the permutation until *r* became *R* or *B* became *b*.

However, when *r* is 0 or *B*, the variance becomes 0 and it is not reasonable to use the criteria for terminating the permutation test. Thus, we adopt a negative binomial distribution. Let *Y* be a positive integer random variable indicating the number of trials and *α*_*e*_=*α*_*p*_×*m*, where *m* is the number of SNP sets. Assuming that the true *p*-value is at most *α*_*p*_ (when *r* is 0) or at least *α*_*e*_ (when *r* is *B*), we attempt to obtain the probability of *B* occurring with *r* and finish the permutation test at *α*_*p*_. Hence, when *r* is 0, if the probability *NB*(*Y*=*B*;*B*,1−*α*_*p*_) is less than *α*_*p*_, which gives an *α*_*p*_ confidence level of $\hat {p}=\alpha _{p}$, the permutation test can be stopped and we obtain $\hat {p}=1/B$. Similarly, when *r* is *B*, if the probability *NB*(*Y*=*B*;*B*,*α*_*e*_) is less than *α*_*p*_, the permutation test can be stopped and we obtain $\hat {p}=1$.

If more precise *p*-values are needed for significant SNP sets, we can ignore the stop criterion if $\hat {p}<\alpha _{p}$ and proceed with *b* permutation tests to obtain the minimal $\hat {p}=1/B$.

### Adaptive SKAT

Our proposed procedure adaptively stops the permutation test when the significance level *α*_*p*_ is outside the *α* confidence interval of the estimated *p*-value using the binomial distributions described in the previous subsection. The proposed procedure is described in Algorithm 1. The following values are taken as input parameters: the significance level *α*_*e*_ (*α*_*p*_=*α*_*e*_/*m*), maximum number of permutation tests *b*, which must be at least 1/*α*_*p*_, and significance interval *α* for the Gaussian distribution. Note that, in practice, we should also set the number of tests performed in the same loop to *M* for computational efficiency. We recommend to set *b*=5/*α*_*p*_,*α*=*α*_*p*_, and *M*=1000 as those used in the Results section.



In practice, when SNPs are grouped at the gene level, the number of SNP sets exceeds 20,000. Although our proposed procedure can handle a few phenotypes on a single processor within a reasonable time, multiple phenotypes and their combinations will entail a huge computational cost. As in many association testing procedures, we therefore recommend using parallel computation to calculate the *p*-value for each SNP set on a different core.

## Results and discussion

We first examine the comparison of power and sample size calculation of SKAT, SKAT-O, and the proposed procedure. In these experiments, according to the SKAT R package and previous literatures [4, 5], we adopted the following settings; we used a numerical matrix of 10,000 haplotypes over a 200,000 Base Pair region, where each row represents a different haplotype and each column represents a different SNP marker. The matrix was generated by the calibration coalescent model (COSI) base on the LD structure of European ancestry [8]. As with the SKAT R package, to evaluate the power of the above methods, we simulated datasets under the alternative model; thus, we repeatedly and randomly selected 5 kb regions from a broader region, and then randomly set causal variants from the rare variants with a minor allele frequency (MAF) of less than 0.05 in each simulation. For generating phenotypes, we considered 20 % of the rare variants were causal variants and 80 % of *β*_*j*_ to be positive and the rest to be negative, and set max effect size as {0.4,0.8,1.2,1.6,2.0}. The results of 1,000 simulations at *α*_*e*_ = {0.01,10^−3^,10^−4^} and the sample size {250,500,750,1,000,1,250,1,500} are summarized in Tables 1, 2, 3, 4 and 5. These results show that the proposed procedure can perform relatively higher power than SKAT and SKAT-O even when the sample size and the effect size are small, and also could retain the competitive power when these are high values, which can achieve type II error of almost 0.2. Even when the phenotype is not according to the idea distribution, the proposed procedure could control the lower type I error than that of SKAT-O.

**Table 1:** The power comparison of SKAT, SKAT-O, and AP-SKAT aimed at testing the association between randomly selected 5 kb regions and continuous traits under the effect size = 0.4

SKAT				SKAT-O			AP-SKAT		
	10^−2^	10^−3^	10^−4^	10^−2^	10^−3^	10^−4^	10^−2^	10^−3^	10^−4^
250	1.47E-2	1.67E-3	1.89E-4	1.48E-2	1.69E-3	1.95E-4	1.51E-2	1.80E-3	2.40E-4
500	2.13E-2	2.90E-3	4.03E-4	2.15E-2	2.92E-3	4.03E-4	2.17E-2	3.07E-3	4.91E-4
750	2.92E-2	4.67E-3	7.35E-4	2.94E-2	4.69E-3	7.38E-4	2.97E-2	4.74E-3	8.37E-4
1000	3.84E-2	6.86E-3	1.24E-3	3.86E-2	6.95E-3	1.25E-3	3.91E-2	7.08E-3	1.33E-3
1250	4.92E-2	9.60E-3	1.92E-3	4.93E-2	9.72E-3	1.92E-3	4.95E-2	9.71E-3	2.05E-3
1500	6.05E-2	1.31E-2	2.81E-3	6.07E-2	1.31E-2	2.82E-3	6.14E-2	1.32E-2	3.05E-3

**Table 2:** The power comparison of SKAT, SKAT-O, and AP-SKAT aimed at testing the association between randomly selected 5 kb regions and continuous traits under the effect size = 0.8

SKAT				SKAT-O			AP-SKAT		
	10^−2^	10^−3^	10^−4^	10^−2^	10^−3^	10^−4^	10^−2^	10^−3^	10^−4^
250	3.83E-2	6.96E-3	1.23E-3	3.85E-2	6.96E-3	1.25E-3	3.91E-2	7.02E-3	1.36E-3
500	8.60E-2	2.22E-2	5.41E-3	8.72E-2	2.23E-2	5.47E-3	8.86E-2	2.23E-2	5.66E-3
750	1.52E-1	4.83E-2	1.45E-2	1.52E-1	4.84E-2	1.46E-2	1.55E-1	4.88E-2	1.52E-2
1000	2.21E-1	8.19E-2	2.96E-2	2.24E-1	8.50E-2	2.99E-2	2.26E-1	8.53E-2	3.02E-2
1250	2.98E-1	1.30E-1	5.10E-2	2.99E-1	1.31E-1	5.29E-2	3.01E-1	1.32E-1	5.37E-2
1500	3.70E-1	1.91E-1	8.11E-2	3.74E-1	1.93E-1	8.28E-2	3.75E-1	1.90E-1	8.49E-2

**Table 3:** The power comparison of SKAT, SKAT-O, and AP-SKAT aimed at testing the association between randomly selected 5 kb regions and continuous traits under the effect size = 1.2

SKAT				SKAT-O			AP-SKAT		
	10^−2^	10^−3^	10^−4^	10^−2^	10^−3^	10^−4^	10^−2^	10^−3^	10^−4^
250	1.01E-1	2.72E-2	7.04E-3	1.01E-1	2.75E-2	7.11E-3	1.03E-1	2.73E-2	7.42E-3
500	2.57E-1	1.04E-1	3.90E-2	2.59E-1	1.05E-1	3.94E-2	2.64E-1	1.06E-1	4.13E-2
750	4.16E-1	2.32E-1	1.07E-1	4.19E-1	2.34E-1	1.09E-1	4.21E-1	2.33E-1	1.12E-1
1000	5.06E-1	3.64E-1	2.18E-1	5.07E-1	3.64E-1	2.20E-1	5.11E-1	3.63E-1	2.18E-1
1250	5.79E-1	4.62E-1	3.36E-1	5.81E-1	4.64E-1	3.39E-1	5.83E-1	4.62E-1	3.37E-1
1500	6.68E-1	5.01E-1	4.29E-1	6.66E-1	5.01E-1	4.32E-1	6.72E-1	5.02E-1	4.28E-1

**Table 4:** The power comparison of SKAT, SKAT-O, and AP-SKAT aimed at testing the association between randomly selected 5 kb regions and continuous traits under the effect size = 1.6

SKAT				SKAT-O			AP-SKAT		
	10^−2^	10^−3^	10^−4^	10^−2^	10^−3^	10^−4^	10^−2^	10^−3^	10^−4^
250	2.19E-1	8.09E-2	2.90E-2	2.21E-1	8.30E-2	2.95E-2	2.24E-1	8.32E-2	2.97E-2
500	4.84E-1	3.05E-1	1.63E-1	4.80E-1	3.04E-1	1.64E-1	4.85E-1	3.01E-1	1.67E-1
750	5.99E-1	4.83E-1	3.69E-1	5.99E-1	4.82E-1	3.71E-1	6.09E-1	4.80E-1	3.66E-1
1000	7.42E-1	5.42E-1	4.88E-1	7.41E-1	5.42E-1	4.89E-1	7.48E-1	5.46E-1	4.87E-1
1250	8.50E-1	6.52E-1	5.14E-1	8.50E-1	6.54E-1	5.13E-1	8.50E-1	6.54E-1	5.16E-1
1500	9.19E-1	7.50E-1	5.93E-1	9.20E-1	7.48E-1	5.90E-1	9.18E-1	7.48E-1	5.94E-1

**Table 5:** The power comparison of SKAT, SKAT-O, and AP-SKAT aimed at testing the association between randomly selected 5 kb regions and continuous traits under the effect size = 2.0

SKAT				SKAT-O			AP-SKAT		
	10^−2^	10^−3^	10^−4^	10^−2^	10^−3^	10^−4^	10^−2^	10^−3^	10^−4^
250	3.77E-1	2.00E-1	8.70E-2	3.82E-1	2.01E-1	8.84E-2	3.83E-1	1.97E-1	8.88E-2
500	6.14E-1	4.89E-1	3.88E-1	6.14E-1	4.90E-1	3.88E-1	6.24E-1	4.89E-1	3.79E-1
750	8.16E-1	6.12E-1	5.01E-1	8.16E-1	6.07E-1	5.01E-1	8.16E-1	6.14E-1	5.02E-1
1000	9.30E-1	7.68E-1	6.10E-1	9.30E-1	7.66E-1	6.11E-1	9.27E-1	7.69E-1	6.12E-1
1250	9.81E-1	8.96E-1	7.48E-1	9.80E-1	8.94E-1	7.42E-1	9.78E-1	8.78E-1	7.42E-1
1500	9.95E-1	9.61E-1	8.58E-1	9.95E-1	9.60E-1	8.59E-1	9.94E-1	9.48E-1	8.49E-1

Additionally, we evaluated the type I error rate of SKAT-O and the proposed procedure when {*β*_1_,…,*β*_*m*_ are 0 and *ε* in Eq. (2) is according to the Student’s t -distribution with 5 degrees of freedom; thus, the distribution of phenotypes is a heavier tailed distribution than the ideal normal one. In this setting, we applied Illumina WGS data for 2,504 samples from 26 populations across Africa, East and South Asia, Europe, and the Americas in the 1000 Genome Project [9] and performed 50 experiments for each sample size of {500,1,000,1,500,2,000}, which are randomly extracted from the data. The results of the number of false positives in using SKAT-O and the proposed procedure are concluded in Table 6 and it indicates that the proposed method can reduce the number of false positives even when the distribution has heavier tails than the normal ones.

**Table 6:** Type I errors of SKAT-O and AP-SKAT to evaluate the inflation of *p*-values using 1000 Genomes Project data under the noises according to the Student’s t -distribution with 5 degrees of freedom

Sample Size	500	1000	1500	2000
SKAT-O	356	178	202	142
AP-SKAT	348	153	189	130

Finally, to validate the proposed approach, we compared the computation times and estimated the *p*-values given by the permutation test (standard procedure) and the adaptive procedure. For this comparison, we prepared genotype data on the previous WGS data from 1000 Genome Project, Illumina Infinium 550 SNP BeadChip for 1,438 samples from the 1958 British Birth Cohort in the Wellcome Trust Case Control Consortium [10], and on the Illumina SNP Chip for 1,397 individuals from 11 populations, including 250 of the original 270 phase I and phase II individuals in the International HapMap Project [11]. Their quantitative phenotype data were synthetically generated according to a Gaussian distribution and SNPs were grouped at the gene level. Note that only those SNPs annotated as ‘High’ and ‘Moderate’ by the SnpEff tool [14] were selected as plausible ones for 1000GP, because WGS data include a lot of less significant SNPs. All SNPs were grouped at the gene level for the data from WTCCC and HapMap. In these experiments, we also consider SNPs with MAF of less than 0.05. The combination of significance levels *α*,*b*, and *M* were set to {0.05,2.5×10^−6^,2.5×10^−11^},{100,1000,…,1.0×10^7^}, and min{*N*/10,10^4^}, respectively. All computations were performed on 800 nodes of an Intel(R) Xeon(R) CPU E5-2680 v2 @ 2.80 GHz (20 cores each) in our supercomputer system.

**Fig. 2:**
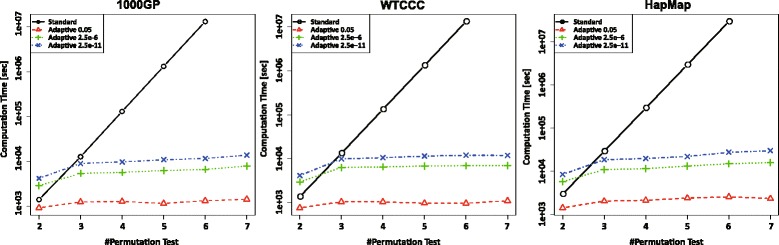
Comparison of computation times between the standard and permutation procedures using 1000 Genomes Project data, WTCCC, and HapMap. *Solid* and *dotted* lines indicate the runtimes of the standard and adaptive procedures, respectively

Figure 2 indicates that the computation time for the standard procedure increases linearly with respect to the number of permutation tests *b*. Hence, the setting with *b*=10^8^ tests was infeasible, even using our supercomputer system. However, the computation time of the adaptive procedure is bounded because the proposed procedure terminates the evaluation of the SNP sets according to a certain criterion. Hence, the computation time of the adaptive procedure depends on the number of significant SNP sets; as only a handful of sets should be selected as significant SNPs, the computational cost is significantly lower than that of the standard procedure. When *b*=100, the computational cost of the adaptive procedure is higher than that of the standard procedure. This is because the adaptive procedure requires additional computation to judge the stop criterion for each *M* loop. However, as *b* should be greater than 1/*α*_*p*_ considering multiple comparisons, the low computational cost when *N*>1.0×10^5^ is more significant.

**Fig. 3:**
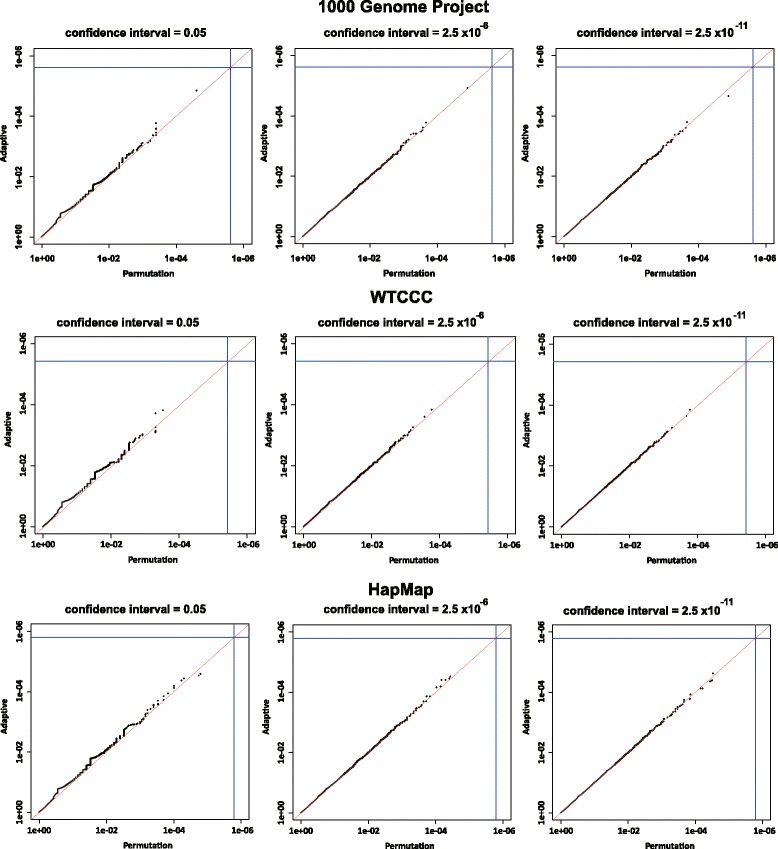
Comparison plot with several confidential intervals using the 1000 Genomes Project data, WTCCC data, and HapMap data. The comparisons of estimated *p*-values for the 1000 Genomes Project data, WTCCC data, and HapMap data by the standard and the adaptive procedures with a significance interval of 0.05,2.5×10^−06^ and 2.5×10^−11^. *Solid* and *dotted* lines are the base line and the Bonferroni corrected significance level (*p*=0.05), respectively. *Circles* indicate the estimated *p*-values of SNP sets by the standard and the adaptive procedures, and the numbers of SNP sets is 20,568,13,397,31,002, respectively. Both the *vertical* and the *horizontal* axes in these figures are logarithmic scale

In Fig. 3, the estimated *p*-values in the adaptive procedure clearly approach those of the standard procedure according to the spread of the confidence interval, and they are almost the same when the confidence interval is lower than 2.5×10^−6^. Even if the confidence interval was set to around 0.05, the tendency of the *p*-values could be observed, enabling us to clarify whether the *p*-values of SNP sets exceeded the threshold value. These results indicate that the proposed procedure can be applied at the whole genome scale to achieve arbitrary confidence levels within a reasonable time.

## Conclusions

In this paper, we proposed a novel rare variant association procedure that can calculate the *p*-values for sets of SNPs within a reasonable time. A comparison experiment showed that the proposed procedure significantly reduced the computational cost while maintaining the estimation quality at predefined significance levels, and can be bounded at a reasonable cost even if we select the highest significance level. This result demonstrates that the proposed procedure is capable of calculating *p*-values of SNP sets for WGS data that cannot be evaluated by the standard permutation procedure. In addition, this procedure can be applied to other common/rare variant association tests [15, 16]. The R code is available at http://nagasakilab.csml.org/data/aSKAT.zip, for which input is either one of PLINK format files or a numeric matrix.

## Availability and requirements

**Project name**: AP-SKAT**Project home page**: http://nagasakilab.csml.org/data/aSKAT.zip**Operating system(s)**: Platform independent**Programming language**: R**Any restrictions to use by non-academics**: Please contact authors for commercial use.
